# Discordant Immune Response with Antiretroviral Therapy in HIV-1: A Systematic Review of Clinical Outcomes

**DOI:** 10.1371/journal.pone.0156099

**Published:** 2016-06-10

**Authors:** Christine Kelly, Katherine M. Gaskell, Marty Richardson, Nigel Klein, Paul Garner, Peter MacPherson

**Affiliations:** 1 Institute of Translation Medicine, University of Liverpool, Liverpool, United Kingdom; 2 Malawi Liverpool Wellcome Trust Clinical Research Program, Blantyre, Malawi; 3 London School of Hygiene and Tropical Medicine, London, United Kingdom; 4 Centre for Evidence Synthesis, Department of Clinical Sciences, Liverpool School of Tropicla Medicine, Liverpool, United Kingdom; 5 Institute of Child Health, University College London, London, United Kingdom; 6 Department of Public Health and Policy, University of Liverpool, Liverpool, United Kingdom; 7 Department of Clinical Sciences, Liverpool School of Tropical Medicine, Liverpool, United Kingdom; 8 North West Public Health England Centre, Liverpool, United Kingdom; Rush University, UNITED STATES

## Abstract

**Background:**

A discordant immune response (DIR) is a failure to satisfactorily increase CD4 counts on ART despite successful virological control. Literature on the clinical effects of DIR has not been systematically evaluated. We aimed to summarise the risk of mortality, AIDS and serious non-AIDS events associated with DIR with a systematic review.

**Methods:**

The protocol is registered with the Centre for Review Dissemination, University of York (registration number CRD42014010821). Included studies investigated the effect of DIR on mortality, AIDS, or serious non-AIDS events in cohort studies or cohorts contained in arms of randomised controlled trials for adults aged 16 years or older. DIR was classified as a suboptimal CD4 count (as defined by the study) despite virological suppression following at least 6 months of ART. We systematically searched PubMed, Embase, and the Cochrane Library to December 2015. Risk of bias was assessed using the Cochrane tool for assessing risk of bias in cohort studies. Two authors applied inclusion criteria and one author extracted data. Risk ratios were calculated for each clinical outcome reported.

**Results:**

Of 20 studies that met the inclusion criteria, 14 different definitions of DIR were used. Risk ratios for mortality in patients with and without DIR ranged between 1.00 (95% CI 0.26 to 3.92) and 4.29 (95% CI 1.96 to 9.38) with the majority of studies reporting a 2 to 3 fold increase in risk.

**Conclusions:**

DIR is associated with a marked increase in mortality in most studies but definitions vary widely. We propose a standardised definition to aid the development of management options for DIR.

## Background

Antiretroviral therapy (ART) substantially reduces the incidence of acquired immunodeficiency syndrome (AIDS) and mortality, with increased CD4 cell count significantly and independently associated with improved prognosis [1–4]. Some patients do not achieve CD4 cell count reconstitution with ART, despite achieving suppression of HIV viral load in the blood [5]. This paradoxical response is referred to by various terms in the literature including discordant immune response (DIR), poor or suboptimal immune reconstitution, incomplete immune recovery or restoration and immunological non-response. Here, we use the term discordant immune response as it was the term most frequently used by the included studies [6–10]. There is currently no agreed case definition for DIR.

Over 13 million people worldwide are on ART, with a further 22 million eligible [11]. Understanding limitations to its success will be critical in improving individual responses to treatment and regimen durability. The 2013 World Health Organization (WHO) consolidated guidelines on treatment of HIV now favour use of HIV viral load monitoring for routine identification of ART treatment failure [12], but CD4 cell counts for patients established on ART remain an important clinical and prognostic tool and are essential for identifying DIR [13, 14].

Much research has focused on CD4 reconstitution on ART, but the mechanisms promoting DIR are not well understood. Damage to CD4 T cells begins prior to ART initiation due to direct effects of the HIV virus on thymic tissue and depletion of progenitor cells[15]. Thymic output may be disproportionally affected in patients who start ART at lower CD4 counts leading to under-reconstitution of naïve CD4 T cells[16, 17]. Lymph node fibrosis is also major feature and correlates with duration of HIV infection prior to ART initiation [18, 19]. Untreated HIV infection leads to a significant activation of the immune system [20], resulting in a cycle of systemic inflammation, persistent T cell activation, exhaustion and death [21–23]. The extent of immune activation at the time of ART initiation is associated with the development of DIR [4, 24] and predicts mortality on ART [25]. HIV induced T cell dysfunction and inflammation are closely related to serious non-AIDS events [20, 26]. Persistent immune activation is often detected despite virologically suppressive ART [27] and can be driven by microbial translocation[28], low level persistent HIV viral replication[29], and latent co-infections such as CMV [30, 31] and tuberculosis [32, 33]. Innate immune cells including monocytes, macrophages and NK cells also perpetuate immune activation, but this axis is more specifically driven microbial translocation, LPS antigenaemia and circulating soluble CD14 and does not necessarily correlate with T cell activation [34–37].

Non-systematic reviews have previously been carried out into aetiologies, prevalence and potential management of DIR [38–44]. However, the literature is heterogeneous and in order to better understand the burden of DIR, we sought to systematically characterise the risk of mortality, AIDS and serious non-AIDS events associated with DIR across the published literature.

## Methods

The study protocol was registered with the Centre for Review Dissemination, University of York (registration number CRD42014010821). The systematic review has been reported in accordance with the PRISMA guidelines [45] (See S1 Checklist).

### Eligibility criteria

#### Participants

Participants were aged 16 years or older and no restrictions were placed on language or geographical region.

Participants with DIR were defined as patients who had been taking ART for at least 6 months and who were virologically suppressed, but had a suboptimal CD4 count according to study definitions. Studies defined a suboptimal CD4 count in terms of either a failure to achieve a pre-specified rise in CD4 count or a pre-specified absolute CD4 value at a specific time point following ART initiation. Virological suppression was defined as at least one single HIV viral load measurement of below 1000 copies/ml after at least 6 months of ART. Studies that did not report on the virological status of the cohort were not included.

#### Outcomes

Studies were included if they estimated the risk of mortality, AIDS or serious non-AIDS events associated with DIR. Studies were included if death was verified by clinician review, tracing or verbal autopsy. AIDS was defined as any illness that met criteria for a WHO stage 4 condition [46]. Serious non-AIDS events were defined as illnesses not included in the WHO Clinical Staging System, and which were non-communicable. These include non-communicable cardiovascular, liver, renal and bone diseases as well as non-AIDS related malignancies. Studies reporting AIDS and serious non-AIDS events were deemed to meet our inclusion criteria if the events had been verified at least by clinician review of participant records.

#### Study design

Studies were eligible for inclusion if they were cohort studies or randomised controlled trials (RCTs). We excluded editorials and comments, case reports and case series, qualitative studies, mathematical modelling studies, and economic analyses.

### Information sources and search methods

We searched the following databases: Cochrane Central Register of Controlled Trials (CENTRAL, in the Cochrane Library issue 1, 2016); MEDLINE (PubMed; 1966 to 31^st^ December 2015); EMBASE (OVID; 1980 to 31^st^ Decemeber 2015). Table 1 shows the search strategy used in Medline (PubMed); this was modified for the other electronic databases.

**Table 1:** Search strategy.

Search	
#16	Search **(#5) AND #15**
#15	Search **(((((((((#6) OR #7) OR #8) OR #9) OR #10) OR #11) OR #12) OR #13) OR #14) OR #15** Field: **Title/Abstract**
#14	Search incomplete CD4* response Field: **Title/Abstract**
#13	Search discordant* Field:Title/Abstract
#12	Search immunovirological discordance* Field: **Title/Abstract**
#11	Search low CD4* Field: **Title/Abstract**
#10	Search insufficient CD4* Field: **Title/Abstract**
#9	Search suboptimal CD4* Field: **Title/Abstract**
#8	Search low responder* Field: **Title/Abstract**
#7	Search suboptimal immune response*[Title/Abstract]
#6	Search **suboptimal immune reconstitution** [**Title/Abstract]**
#5	Search **(#1) AND #4**
#4	Search **(#2) OR #3**
#3	Search **(antiretroviral[Title/Abstract]) OR ART[Title/Abstract]**
#2	Search **antiretroviral therapy [Title/Abstract]**
#1	Search **"HIV Infections"[Mesh]**

### Study Selection and Data Collection

Titles of studies identified from the database search were independently reviewed by two authors (CK and KG) and were excluded if the study was unrelated to the review subject. Remaining studies underwent independent abstract review and then full text review by the same two reviewers. Pre-piloted data extraction forms were independently applied to all studies that underwent full text review. Where disagreement occurred, a consensus was reached by discussion or a third reviewer was consulted (PM). Where outcome data were not reported, the lead study author was contacted.

### Risk of bias

Risk of bias assessment was based on the Cochrane Tool for Assessing Risk of Bias in Cohort Studies [47]. The Cochrane Tool for Assessing Risk of Bias in Randomised Control Trials was not used because no RCTs targeting DIR were identified that met the inclusion criteria. Potential sources of bias were assessed in three domains: ‘study design’; ‘comparability’; and ‘assessment of outcomes’.

The study design domain assessed whether participants were selected to be representative of adults on ART, and if there were clear selection criteria for those with and without DIR. Studies with more stringent selection criteria based on, for example, frequency of CD4 and viral load monitoring or attendance at routine clinics prior to enrolment, were deemed to be at a high risk of bias because they might exclude populations at higher risk of DIR and therefore were not representative of the entire population of patients with DIR. The comparability domain assessed if patients with and without DIR were managed according to the same standardised protocol and if outcomes were reported after appropriate adjustment for potential confounding variables. For the assessment of outcomes domain, outcomes had to be measured using clinician review, case note review, verbal autopsy or autopsy. Studies that did not report at least one of these methods were deemed high risk of bias for this category. The minimum acceptable follow-up period was one year as this is the highest risk period for adverse clinical outcomes post ART initiation [48].

An overall risk of bias assessment was made for each individual domain. A domain would be classified as high risk of bias if any one question within it failed the specified criteria. Where insufficient information had been reported in a study to make a judgement on the risk of bias, that question was recorded as unclear. ‘Unclear’ and ‘high risk’ categories were then combined for the purposes of analysis [49].

### Summary measures and synthesis of results

For each study, the proportion of participants with and without DIR who died, and/or experienced an AIDS-related, or serious non-AIDS-related event were estimated. Risk ratios and 95% confidence were extracted from the manuscript or calculated. A meta-analysis with pooled effect estimates were planned but could not be carried out due to considerable variation in both DIR definition and length of follow-up.

## Results

### Study selection

**Fig 1:**
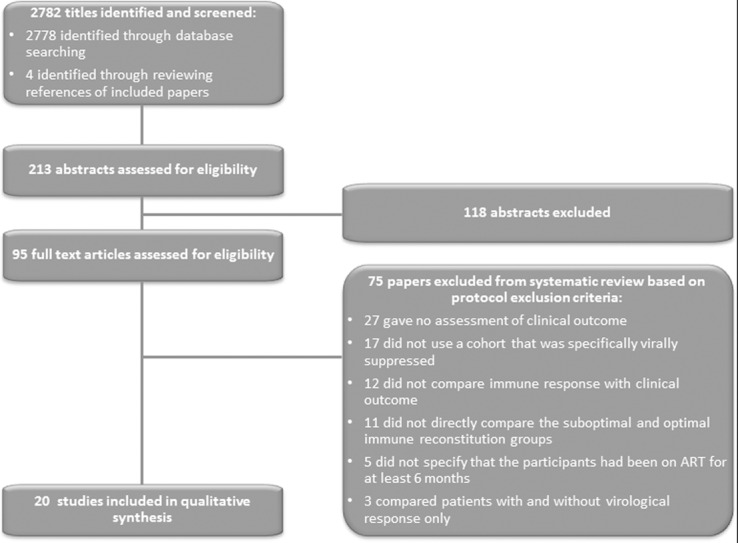
Flow of paper selection from those identified following literature search through to inclusion.

2782 study titles were identified by the search. Twenty studies met inclusion criteria for full-text review (Fig 1). The two most common reasons for exclusion were that the study did not report a clinical outcome (36%) and the cohort was not virally suppressed (23%). Authors from the study by Young *et al* were contacted to clarify if participants had been on ART for at least 6 months but the data were no longer available and so the study was excluded.

### Study characteristics

Twenty studies were included [4, 6–9, 50–64], all of which were cohort studies. For three studies, the cohort was established *de novo* to investigate the effects of DIR on clinical outcomes [4, 51, 58, 64]. One study analysed DIR and outcomes in the control arm of an RCT assessing ART regimes for individuals initiating ART [50]. The remaining 14 studies conducted secondary analysis of existing datasets comprising of national or international cohorts of HIV infected patients who had data collected prospectively and systematically during routine clinical care [6–9, 52–57, 59–63].

Seventeen studies recruited participants from HIV care clinics. Five studies included participants from resource-limited countries [4, 9, 58, 63, 64]: three from countries with a generalised HIV epidemic (Uganda, South Africa) [4, 58, 64]; one from Senegal [63]; and one from an international collaboration of both low-, and middle-income countries [9]. Participants were ART-naïve in 16 studies [4, 6–9, 50, 51, 53, 55–60, 63, 64] whereas four studies included patients who were ART naïve and experienced [52, 54, 61, 62].

For included studies, the median proportion of male participants ranged from 31%– 100% and median age ranged from range 34 to 43 years (Table 2). Median CD4 cell count at ART initiation was reported for 15 studies and ranged from 80–221 cells/mm^3^. Median HIV viral load at ART initiation was reported for 10 studies and ranged from 4.5 log_10_−5.1 log_10_ copies/ml. The threshold for defining virological suppression ranged from <50 copies/ml to <1000 copies per ml. Participant follow-up ranged from 1 to 7 years.

**Table 2:** Description of 20 included studies.

**Study author**	**Study design**	**Year of publication**	**Median Duration of follow-up**	**Country**	**Setting**	**Relevant Outcomes examined**	**ART naïve?**	**%Male**	**Median age (years)**	**Median CD4 (cells/uL) at ART initiation**	**Median HIV VL (log10 copies/mL) at ART initiation**
BAKER [50]	Control arm of ART RCT	2008	5 years	USA	Community, 80 sites	Predictors and clinical outcomes of patients with DIR	Yes	80	39	221	5.0
BATISTA [63]	Established HIV cohort^2^	2015	7 years	Senegal	HIV care clinic	Frequency and risk factors for DIR, and incidence of OI and death	Yes	35	40	Not reported	Not reported
DRONDA [51]	Prospective cohort study^1^	2002	3 years	Spain	HIV care clinic	Immunologic and clinical outcomes of patients with DIR	Yes	74	36	196	5.0
ENGSIG [52]	Established HIV cohort^2^	2010	4.7 years	Denmark	HIV care clinics, 8 sites	Predictors and mortality of patients with DIR	No	78	43^5^	Not reported	Not reported
FALSTER [53]	Established HIV cohort^2^	2008	5.4 years	Australia	HIV care clinics, number of sites not reported	Prevalence of DIR, and and clinical outcomes	Yes	93^5^	Not reported	Not reported	Not reported
GILSON [6]	Established HIV cohort^2^	2010	3 years	UK	HIV care clinics, 10 sites	Predictors and clinical outcomes	Yes	75	37	170	5
**Study author**	**Study design**	**Year of publication**	**Median Duration of follow-up**	**Country**	**Setting**	**Relevant Outcomes examined**	**ART naïve?**	**%Male**	**Median age (years)**	**Median CD4 (cells/uL) at ART initiation**	**Median HIV VL (log10 copies/mL) at ART initiation**
GRABAR [54]	Established HIV cohort^2^	2000	18 months	France	HIV care clinics, 68 sites	Clinical outcomes of patients with DIR	No	79	37	150	4.54
GUTERRIEZ [55]	Established HIV cohort^2^	2008	2.3 years	Spain	HIV care clinics, 10 sites	Predictors and clinical outcomes of patients with DIR	Yes	75	37	160	5.0
HUNT [4]	Prospective cohort study^1^	2011	2 years	Uganda	HIV care clinic	Mortality according to CD4 account^3^	Yes	30	34	135	5.1
KAUFMANN [56]	Established HIV cohort^2^	2004	5 years	Switzerland	HIV care clinics, number of sites not reported	Predictors and clinical outcomes of patients with DIR	Yes	74	38	180	4.9
LOUTFY [57]	Established HIV cohort^2^	2010	2.7 years	Canada	HIV care clinics, 9 sites	Clinical outcomes of patients with DIR	Yes	83	40	180	5.0
MOORE [7]	Established HIV cohort^2^	2005	3.7 years	Canada	HIV care clinic	Predictors and mortality in patients with DIR	Yes	77^5^	39	199	Not reported
NAKANJAKO [58]	Prospective cohort study^1^	2008	1.8 years	Uganda	HIV care clinic	Prevalence of DIR and clinical outcomes	Yes	31	38	98	Not reported
**Study author**	**Study design**	**Year of publication**	**Median Duration of follow-up**	**Country**	**Setting**	**Relevant Outcomes examined**	**ART naïve?**	**%Male**	**Median age (years)**	**Median CD4 (cells/uL) at ART initiation**	**Median HIV VL (log10 copies/mL) at ART initiation**
NICASTRI [61]	Established HIV cohort^2^	2005	3.7 years	Italy	Hospital, 63 sites	Immunologic and clinical outcomes	No	72	35	185	4.78
PACHECO [59]	Established HIV cohort^2^	2009	6 years	Spain	Hospital, 10 sites	CD4 count recovery, predictors and mortality in patients with DIR	Yes	32^5^	Not reported	Not reported	Not reported
TAKUVA [64]	Prospective cohort study^1^	2014	2 years	South Africa	HIV care clinic, 1 site	Mortality and AIDS associated with DIR	Yes	36	39	80	Not reported
TAN [8]	Established HIV cohort^2^	2008	3.2 year	USA	HIV care clinic	Clinical outcomes in patients with DIR	Yes	76	38	213	5.4
TAIWO [62]	Established HIV cohort^2^	2009	Not reported	USA	HIV care clinics, 4 sites	Clinical outcomes in patients with DIR	No	100	42	Not reported	Not reported
TUBOI [9]	Established HIV cohort^2^	2010	1 year	Multi-centre^4^	HIV care clinics, 31 centres	Mortality in patients with DIR	Yes	39	34	100	Not reported
ZOUFALY [10]	Established HIV cohort^2^	2010	3.8 years	Germany	HIV care clinics, 11 sitess	Predictors and clinical outcomes in patients with DIR	Yes	77	39	80	Not reported

ART = anti-retroviral therapy, cART = combination anti-retroviral therapy, DIR = discordant immune response, VL = Viral load, PI = Protease inhibitor, NRTI = nucleoside reverse transcriptase inhibitor, DDI = didanosine, TDF = tenofovir, LMIC- = Low and middle income countries.

^1^ Patients are enrolled specifically for the aims of the current study.

^2^ Retrospective analysis of prospectively collected data.

^3^Analysis of clinical outcomes in DIR is a secondary analysis in this study.

^4^Includes countries from Africa, South America and Asia.

^5^Not reported for entire cohort therefore median value from optimal immune response group reported.

### Risk of bias

Two (10%) studies had a high risk of bias in study design; 6 (30%) in comparability; and 11 (55%) in assessment of outcomes (Table 3).

**Table 3:** Risk of bias assessment for 20 included studies.

Study	Study design			Comparability			Assessment of outcomes					Overall risk of bias
	Were participants selected to be representative of the wider population?	Were there clear selection criteria for those with and without DIR?	Risk of bias	Are patients with and without DIR managed to standardised protocol?	Are outcomes reported after adjustment for important confounding variables?	Risk of bias	Were procedures for measuring outcome sufficient?	Was follow-up long enough for outcome detection?	Were incomplete outcome data adequately assessed?	Are outcomes reported in full and not selectively reported?	Risk of bias	
BAKER [50]	Yes	Yes	**Low**	Yes	Unclear	**High**	Yes	Yes	Unclear	Yes	**High**	**High**
BATISTE [63]	Yes	Yes	**Low**	Yes	Yes	**Low**	Yes	Yes	No	Yes	**High**	**High**
DRONDA [51]	Yes	Yes	**Low**	Yes	No	**High**	Yes	Yes	Yes	Yes	**Low**	**High**
ENGSIG [52]	No	Yes	**High**	Yes	No	**High**	Yes	Yes	Unclear	Yes	**High**	**High**
FALSTER [53]	Yes	Yes	**Low**	Yes	No	**High**	Yes	Yes	Unclear	Yes	**High**	**High**
GILSON [6]	Yes	Yes	**Low**	Yes	Yes	**Low**	Yes	Yes	Unclear	Yes	**High**	**High**
GRABAR [54]	Yes	Yes	**Low**	Yes	Yes	**Low**	Yes	Yes	Unclear	Yes	**High**	**High**
GUTERRIEZ [55]	Yes	Yes	**Low**	Yes	Yes	**Low**	Yes	Yes	Yes	Yes	**Low**	**Low**
HUNT [4]	Yes	Yes	**Low**	Yes	Yes	**Low**	Yes	Yes	Yes	Yes	**Low**	**Low**
KAUFMANN [56]	No	Yes	**High**	Yes	No	**High**	Unclear	Yes	No	Yes	**High**	**High**
LOUTFY [57]	Yes	Yes	**Low**	Yes	Yes	**Low**	Unclear	Yes	No	Yes	**High**	**High**
MOORE [7]	Yes	Yes	**Low**	Yes	Yes	**Low**	Yes	Yes	Yes	Yes	**Low**	**Low**
NAKANJAKO [58]	Yes	Yes	**Low**	Yes	No	**High**	Yes	Yes	No	Yes	**High**	**High**
NICASTRI [61]	Yes	Yes	**Low**	Yes	Yes	**Low**	Yes	Yes	Yes	Yes	**Low**	**Low**
PACHECO [59]	Yes	Yes	**Low**	Yes	Yes	**Low**	Yes	Yes	No	Yes	**High**	**High**
TAKUVA [64]	Yes	Yes	**Low**	Yes	Yes	**Low**	Yes	Yes	Yes	Yes	**Low**	**Low**
TAN [8]	Yes	Yes	**Low**	Yes	Yes	**Low**	Yes	Yes	Yes	Yes	**Low**	**Low**
TAIWO [62]	Yes	Yes	**Low**	Yes	Yes	**Low**	Yes	Unclear	Unclear	Yes	**High**	**High**
TUBOI [9]	Yes	Yes	**Low**	Yes	Yes	**Low**	Yes	Yes	Yes	Yes	**Low**	**Low**
ZOUFALY [10]	Yes	Yes	**Low**	Yes	Yes	**Low**	Yes	Yes	Unclear	Yes	**High**	**High**

For the study design domain, Engsig *et al* required a viral load of <50 copies /ml for more than three consecutive years before the start of the DIR observation period [52], and Kaufmann *et al* required a viral load of <1000 copies/mL during the entire 5-year observation period [56]. The frequency of visits these criteria would require may have excluded patients at higher risk of DIR. All studies detailed clear selection criteria for participants with and without DIR.

For the comparability domain, participants with and without DIR were managed according to the same treatment protocols for all studies but 6 studies did not appropriately evaluate the effects of confounders on outcomes [50–53, 56, 58].

For the assessment of outcomes domain, two studies gave no information on how deaths [56, 57] or AIDS events [57] were ascertained. Eleven studies did not describe how missing data were handled [6, 50, 52, 53, 56–60, 62, 63].

### Definition of DIR

Definitions of DIR varied significantly and were classified into two categories: a failure to achieve a prespecified absolute CD4 count at a predefined time point; or a failure to achieve a prespecified rise in CD4 count from baseline at a predefined time point (Table 4). Five studies explored several potential definitions of DIR [6, 10, 57, 58, 64].

**Table 4:** Effect of DIR on rate of clinical outcomes, according to DIR definitions, for 20 studies reporting clinical outcomes.

Definition of discordant immune response	First Author	HIV viral load cut off	Number virologically suppressed	Number virologically suppressed with DIR	Effect of DIR on risk of Mortality			Effect of DIR on risk of AIDS			Effect of DIR on risk of AIDS or mortality		
					DIR number of participants (%)	IR number of participants (%)	Risk ratio (min CI–max CI)	DIR number of participants (%)	IR number of participants (%)	Risk ratio (min CI–max CI)	DIR number of participants (%)	IR number of participants (%)	Risk ratio (min CI–max CI)
Failure to achieve rise in CD4 count of > = 50 cells/mm^3^ at 6 months after ART initiation	MOORE [7]	<500 at 6 months	1084	235	53 (22.6)	61 (7.2)	3.14 (2.24–4.40)	NR	NR	NR	NR	NR	NR
	TAN [8]	Undetected at 6 months	320	35	4 (11.4)	11 (3.9)	2.96 (1.00–8.80)	6 (17.1)	30 (10.5)	1.63 (0.73–3.54)	NR	NR	NR
	TUBOI [9]	<500 at 6 months	6234	1260	23 (4.5)	51 (1.0)	1.78 (1.09–2.90)	NR	NR	NR	NR	NR	NR
Failure to achieve rise in CD4 count of > = 50 cells/mm^3^ at 8 months after ART initiation	BAKER [50]	<400 at 8 months	850	149	7 (4.7)	19 (2.7)	1.73 (0.74–4.03)	16 (10.7)	33 (4.7)	2.28 (1.29–4.02)	NR	NR	NR
Failure to achieve rise in CD4 count of > = 50 cells/mm^3^ at 12 months after ART initiation	GRABAR [54]	<1000 at 6 months	1486	387	NR	NR	NR	NR	NR	NR	37 (9.6)	51 (4.8)	1.99 (1.33–2.99)
	GUTERRIEZ [55]	<500 throughout follow-up	650	108	8 (7.4)	10 (1.8)	4.01 (1.62–9.94)	3 (2.8)	15 (2.8)	1.00 (0.30 -–3.41)	NR	NR	NR
Failure to achieve rise in CD4 count of > = 100 cells/mm^3^ at 12 months after ART initiation	DRONDA [51]	<50 quarterly for 2 years	288	76	NR	NR	NR	NR	NR	NR	7 (9.2)	40 (18.9)	0.86 (0.41–1.80)
	NAKANJAKO [58]	<400 quarterly for 2 years	339	151	NR	NR	NR	14 (9.3)	9 (4.8)	1.94 (0.86–4.35)	NR	NR	NR
Failure to achieve rise in CD4 count of > = 100 cells/mm^3^ at 12 months after ART	NICASTRI [61]	<500 at 12 months	1117	336	NR	NR	NR	NR	NR	NR	Not reported	Not reported	Odds ratio 2.32 (1.36–3.95)
Failure to achieve rise in CD4 count of > = 100 cells/mm^3^ at 8 months after ART initiation	GILSON [6]	<50 twice over one year	2584	571	26 (4.6)	24 (2.0)	2.29 (1.33–3.97)	15 (2.6)	33 (2.8)	0.96 (0.53–1.76)	NR	NR	NR
Failure to achieve an absolute CD4 count of > = 174cells/mm^3^ at 6 months after ART initiation^1^	HUNT [4]	<1000 throughout follow-up	451	107	3 (2.8)	6 (1.7)	1.00 (0.26–3.92)	NR	NR	NR	NR	NR	NR
Failure to achieve an absolute CD4 count of > = 350 cells/mm^3^ at 9 months after ART initiation	FALSTER [53]	<400 twice over one year	292	83	NR	NR	NR	NR	NR	NR	14 (3.5)	35 (2.2)	2.06 (0.89–4.79)
Failure to achieve an absolute CD4 count of > = 200 cells/mm^3^ at 12 months after ART	ZOUFALY [10]	<50 throughout follow-up	1085	248	NR	NR	NR	18 (7.3)	11 (1.3)	2.70 (1.29–5.66)	NR	NR	NR
	LOUTFY [57]	<50 throughout	2028	404	NR	NR	NR	NR	NR	NR	14 (3.5)	35 (2.2)	1.61 (0.87–2.96)
Failure to achieve an absolute CD4 count of > = 250 cells/mm^3^ at 22 months after ART initiation	PACHECO [59]	<1000 quarterly for 2 years	147	40	5 (12.5)	4 (3.7)	3.23 (0.89–11.77)	NR	NR	NR	NR	NR	NR
Failure to achieve an absolute CD4 count of > = 200 cells/mm^3^ at 36 months after ART initiation	ENGSIG [52]	<50 over 3 years	291	55	11 (20)	11 (4.7)	4.29 (1.96–9.38)	NR	NR	NR	NR	NR	NR
Failure to achieve an absolute CD4 count of > = 500 cells/mm^3^ at 60 months after ART	KAUFMANN [56]	<1000 over 5 years	293	105	22 (21.0)	18 (9.6)	2.19 (1.23–3.89)	NR	NR	NR	NR	NR	NR

NR not reported. DIR discordant immune response IR concordant immune response. VL viral load.

Eleven studies defined DIR based on a failure to achieve a rise in CD4 from ART initiation [6–9, 50, 51, 54, 55, 58, 61, 63] and used CD4 count thresholds of a failure to achieve a rise of at least 50 cells/ mm^3^ at 6 months [7–9, 63]; at least 100 cells/ mm^3^ at 12 months [51, 58]; at least 50 cells/ mm^3^ at 12 months [54, 55]; at least 50 cells/ mm^3^ at 8 months [50]; and at least 100 cells/ mm^3^ at 8 months [6].

Nine studies defined DIR based on a failure to achieve an absolute CD4 count at a predefined time point [4, 10, 52, 53, 56, 57, 59, 62, 64] and used the following CD4 count thresholds: 200 cells/ mm^3^ at 6 months[64], 200 cells/ mm^3^ at 12 months [10, 57]; of 350 cells/ mm^3^ at 9 months [53]; of 250 cells/ mm^3^ at 22 months [59]; 200 cells/ mm^3^ at 36 months [52]; and 500cells/ mm^3^ at 60 months [56]. Hunt and colleagues described mortality according to tertiles of CD4 counts in patients with viral suppression rather than use a single definition for DIR [4] so we compared mortality in the highest tertile (>177 cells/mm^3^) to the lowest tertile (<95 cells/mm^3^), using the lowest tertile of CD4 counts as the ‘DIR group’.

HIV VL cut offs used by studies to define virological suppression were as follows: 50 copies/ml (seven studies) [6, 10, 51, 52, 57, 62, 63]; 400 copies/ml (four studies) [50, 53, 58, 64]; 500 copies/ml (four studies) [7, 9, 55, 61]; and 1000 copies/ml (four studies) [4, 54, 56, 59]. One study only reported using an ‘undetectable’ VL [8]. The time period for the definition of virological suppression was as follows: a one off cut off point between 6 months to a year post ART (eight studies) [7–9, 50, 54, 61, 63, 64]; two measurements over one year (two studies) [6, 53]; quarterly measurements for 2 years (two studies) [51, 58, 59], and quarterly measurements throughout the period of follow-up (seven studies) [4, 10, 55–57, 62, 65].

### Effect of DIR on risk of mortality

**Fig 2:**
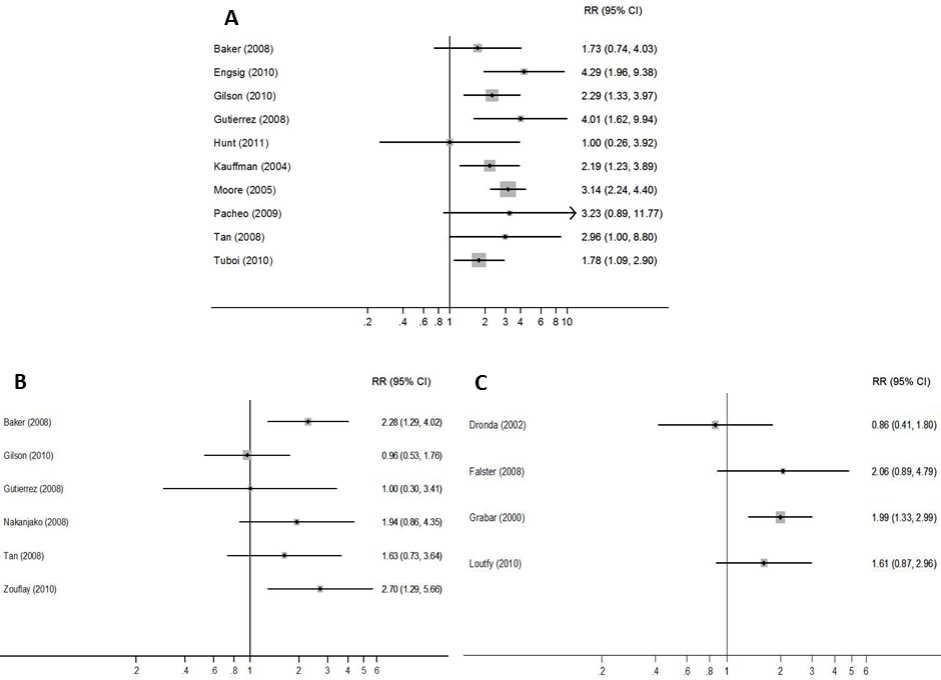
**Forest plot showing risk of clinical outcomes for patients with DIR across those studies reporting each outcome: A) Mortality B) AIDS events C) Combined mortality and AIDS events**.

The risk of mortality ranged between 3% to 23% for patients with DIR and 1% to 7% for patients without DIR, over a median follow-up time of 2 years and 3.7 years respectively. Ten studies estimated the effect of DIR on mortality [4, 6–9, 50, 52, 55, 56, 59] (Table 4). Risk ratios ranged between 1.00 (95% CI 0.26–3.92) and 4.29 (95% CI 1.96–9.38). Six of nine studies showed a significantly higher risk of mortality in participants with DIR compared to participants without DIR [6–9, 55, 56] (Fig 2A). Two studies reported on the absolute risk of mortality in participants with DIR in resource-limited settings [4, 9], with Tuboi *et al* finding DIR to be significantly associated with an increased risk of death.

Ten studies reported the incidence of mortality in participants with DIR (Table 5). Six found the incidence of mortality to be significantly higher in participants with DIR compared to participants without DIR [6–9, 55, 64]. Incidence rate ratios ranged from 1.78 (95% CI 1.09–2.90) to 4.01 (95% CI 1.62–9.94). One study from a sub-Saharan Africa setting (South Africa) reported rates of mortality and found an IRR of 1.78 (1.09–2.90). No differences in risk of mortality were found according to DIR defined as absolute or rise, CD4 count cut off or time period post ART initiation.

**Table 5:** Effect of DIR on rate of clinical outcomes, according to DIR definitions, for 10 studies reporting incidence data.

Definition of discordant immune response *(time periods are length of time following ART initiation)*	First Author	HIV viral load cut off	Number virologically suppressed	Number virologically suppressed with DIR	Effect of DIR on rate of Mortality			Effect of DIR on rate of AIDS			Effect of DIR on rate of AIDS or mortality		
					DIR number of participants (per 100py)	IR number of participants (per 100 py)	Incidence rate ratio (min CI–max CI)	DIR number of participants (per 100 py)	IR number of participants (per 100 py)	Incidence rate ratio (min CI–max CI)	DIR number of participants (per 100 py)	IR number of participants (per 100 py)	Incidence rate ratio (min CI–max CI)
Failure to achieve rise in CD4 count of > = 50 cells/mm^3^ after 6 months	BATISTA [63]	<50 at 6 months	657	102	NR	NR	NR	NR	NR	NR	47 (9.8)	202 (7.8)	1.21 (0.85–1.72)
	MOORE [7]	<500 at 6 months	1084	235	53 (5.7)	61 (1.8)	3.2 (3.9–12.7)	NR	NR	NR	NR	NR	NR
Failure to achieve an absolute CD4 count of > = 200 cells/mm^3^ after 6 months	TAKUVA [64]	<400 at 6 months	4129	NR	NR	NR	2 (1.44–2.79)	NR	NR	1.67 (1.27–2.21)	NR	NR	NR
Failure to achieve rise in CD4 count of > = 100 cells/mm^3^ after 8 months	GILSON [6]	<50 twice over one year	2584	571	26 (3.5)	24 (0.5)	7.00 (3.9–12.7)	15 (2.0)	33 (0.7)	2.9 (1.4–5.4)	NR	NR	NR
Failure to achieve rise in CD4 count of > = 50 cells/mm^3^ after 12 months	GRABAR [54]	<1000 at 6 months	1486	387	NR	NR	NR	NR	NR	NR	37 (6.6)	51 (1.8)	3.7 (2.3–5.7)
Failure to achieve an absolute CD4 count of > = 200 cells/mm^3^ after 12 months	ZOUFALY [10]	<50 throughout	1085	248	18 (4.4)	11 (1.6)	2.8 (1.2–6.4)	NR	NR	NR	NR	NR	NR
	LOUTFY [57]	<50 throughout	2028	404	NR	NR	NR	NR	NR	NR	14 (1.1)	35 (0.8)	1.4 (0.7–2.6)
Failure to achieve an absolute CD4 count of > = 250 cells/mm^3^ after 22 months	PACHECO [59]	<1000 quarterly for 2 years	147	40	5 (2.4)	4 (0.7)	3.2 (0.70–16.4)	NR	NR	NR	NR	NR	NR
Failure to achieve an absolute CD4 count of > = 200 cells/mm^3^ after 36 months	ENGSIG [52]	<50 over 3 years	291	55	26 (3.5)	24 (0.5)	4.4 (1.7–11.3)	NR	NR	NR	NR	NR	NR
Failure to achieve an absolute CD4 count of > = 200 cells/mm^3^ after 6 months	TAIWO [62]	<50 biannually throughout	NR	NR	NR	NR	5.96 (0.40–87.8)	NR	NR	HR 22.8 (1.89–275)	NR	NR	HR 10.7 (1.65–70)

NR not reported. DIR discordant immune response IR concordant immune response. VL viral load. py person years HR hazard ratio

### Effect of DIR on risk of AIDS and serious non-AIDS events

Six studies reported AIDS events [6, 8, 10, 50, 55, 58]. The risk ratio for associations between DIR and AIDS events ranged from 0.96 (95% CI 0.53–1.76) to 2.70 (95% CI 1.29–5.66) (Fig 2B). One of these reported AIDS events in a low resource setting (Uganda) with a risk ratio of 1.94 (0.86–4.35). Five studies reported combined AIDS events or mortality [51, 53, 54, 57, 61] and risk ratios ranged from 0.86 (95% CI 0.41–1.80) to 2.06 (95% CI 0.89–4.79) (Fig 2C). One study from a low resource setting (Senegal) reported an incidence rate ratio of 1.21 (0.85–1.72).

Four studies detailed AIDS events. The most commonly reported pathologies were oesophageal candidiasis, tuberculosis, AIDS related cancers, *pneumocystis jirovecii* and bacterial pneumonia [50, 55, 58, 60]. Only Baker *et al* included serious non-AIDS events [50], reporting events in eight of 143 patients (5.6%) with DIR compared to 31 of 671 patients (4.6%) without.

## Discussion

The main finding of this review was that we found definitions used to categorise DIR varied widely, with 14 different definitions used in the 20 included studies. This greatly limits the ability to draw conclusions about clinical burden in this patient cohort. This paper has synthesised existing data and suggested the definition for DIR to be a rise of less than 50 cells/uL at 6 months following ART initiation in those who have achieved virological suppression but with a CD4 count of less than 350 cells/uL. This provides a starting point for the development of consensus within the field. For the majority of included studies, mortality in patients with DIR was two to three times higher than in those with a satisfactory immune response. To our knowledge, this is the first review to systematically examine the fate of adults with DIR. Two further important gaps in the literature were identified: the large majority of current data reports on cohorts from high income countries; and only one study reported on the burden of serious non-AIDS events. Both the clinical burden of DIR in low income settings and the global burden of serious non-AIDS events remain unclear. It is not possible to draw comparisons between the risks associated with DIR in low resource to high resource settings with the current literature.

Although pooled meta-analysis was inhibited by heterogeneity in length of follow-up, mortality rates remain substantially and significantly elevated in patients with DIR in studies that reported rates adjusted for time. The relationship between DIR and AIDS is less clear and may be complicated by challenges in diagnosing or reporting AIDS conditions. Alternatively, other conditions such as serious non-AIDS events may be contributing to mortality and this warrants further investigation.

The scope of this review was also limited by heterogeneity in DIR definitions. In order to address this, we advocate the use of a standard definition. To define DIR based on a failure to achieve a rise in CD4 from baseline is more reflective of the amount of time spent at a lower CD4 count, which is an important predictor of poor outcomes [14]. In contrast, an absolute CD4 count at a given time point may only tell us about that point in time, when other factors such as co-existing infections may be affecting the CD4 count. The expected rate of CD4 reconstitution following ART initiation is 20 to 30 cells per month in the first 6 months and then 5 to 10 cells per month between 6 months and 24 months [66, 67]. Therefore, when choosing a time point to measure DIR, we believe that 6 months after ART initiation is logical.

Many studies included in this review based their definition of DIR on a failure to achieve a rise of 50 cells/uL at 6 months post ART initiation. Whilst this is a relatively strict CD4 cut off, these studies still reported a high proportion of virologically-suppressed patients with DIR. We would therefore recommend defining DIR as a rise of less than 50 cells/uL at 6 months following ART initiation in patients who have achieved virological suppression. This definition has the benefit of identifying a high risk group of patients early on in the course of their ART management to allow for increased benefit of any potential intervention. It is logical that this definition would only apply to those commencing ART with a CD4 <350 cells/uL so as not to over diagnose DIR in a population starting with higher CD4 counts. The heterogeneity in definitions for DIR and outcome measures means that it is not currently possible to compare the utility of definitions to predict clinical outcomes. We recommend further studies to clinically validate a standardised definition.

This review should be interpreted in the light of several limitations. Firstly, the majority of studies were carried out using data collected from ongoing multicentre cohort studies, meaning cohorts are likely to be highly selected in terms of laboratory monitoring and attending follow-up visits. This limits the generalizability of the studies, and may mean that the risk of adverse clinical outcomes in individuals with DIR could be underestimated. Secondly, the HIV viral load limit defining virological suppression varied across studies. However, it remains unclear whether differences in viral load below 1000 copies/ml are biologically significant [68]. Lastly, individual studies did not distinguish between early mortality in patients starting ART with advanced immunosuppression and long term mortality due to poor immune reconstitution. This could be addressed in future studies.

There are currently no effective therapeutic options to reduce the excess mortality associated with DIR and further research is required. One approach under evaluation is to target underlying drivers of immune activation and inflammation. The addition of raltegravir to standard two class regimes at ART initiation has the aim of decreasing viral set point but as yet only two small studies have shown any effect on immune responses [69, 70]. Similarly a recent trial with valganciclovir to tackle ongoing CMV replication failed to show any improvement in CD4 count [71]. Although probiotics can improve the systemic pro-inflammatory profile, there is no evidence that this can improve CD4 counts [72]. To address generalised inflammation, anti-inflammatory agents such as statins and anti-rheumatic agents have been tested [73, 74]. Whilst statins reduced peripheral immune cell activation, there is no evidence that they can improve CD4 T cell count. Studies investigating the role of quinolones in reducing HIV related immune activation have shown only small decreases in inflammatory markers [75]. Immunomodulatoy agents such as IL-2 have shown limited success [76, 77] and current focus is being placed on IL-7 therapy [78, 79] with several ongoing trials in progress. Lastly, agents aimed at stimulating thymic output have also been tested in early studies [80, 81].

Practical management options may be more accessible in the short term. Standardised guidelines could recommend continuation of prophylactic therapies such as co-trimoxazole and isoniazid for patients with DIR, or could prompt investigation for subclinical opportunistic infections such as tuberculosis and CMV. Although prevention of DIR through early diagnosis of HIV infection and prompt treatment with ART is likely the most effective intervention [82–85], a large proportion of patients worldwide continue to present with advanced HIV infection [86, 87].

This systematic review highlights that a wide range of definitions have been used to characterise clinical outcomes in patients with DIR. These patients are at an increased risk of mortality and are in need of special attention, including integration into HIV clinical trials. We have suggested a definition for DIR based on the limited available data in order to help begin the process of arriving at a consensus definition that could be used to guide clinical care and in future research. We recommend that further studies validate this definition for DIR to aid the development of consensus guidelines.

## Supporting Information

S1 Checklist PRISMA Guidelines. (DOC)
